# Replication Timing Aberration of *KIF14* and *MDM4* / ***PI3KC*** 2 *β* Alleles and Aneuploidy as Markers of Chromosomal Instability and Poor Treatment Response in Ewing Family Tumor Patients

**DOI:** 10.1055/s-0043-1768238

**Published:** 2023-04-21

**Authors:** Fernanda Rocha Rojas Ayala, Jeffrey William Martin, Carmen Silvia Bertuzzo

**Affiliations:** 1 Department of Medical Genetics, Unicamp University, Oncogenetics Service at Clinics Hospital, Campinas, SP, Brazil; 2 Department of Medicine, Toronto University, Toronto, Ontario, Canada; 3 Department of Genetics and Genomics, at Unicamp University, Oncogenetics Services at Clinics Hospital, Campinas, SP, Brazil

**Keywords:** replication timing, aneuploidy, chromosomal instability, poor treatment response, Ewing sarcoma

## Abstract

Replication timing of allelic gene pairs is strictly regulated according to expression, genome stability, and epigenetic changes, and tumorigenesis may be associated with changes in the allelic replication in various tumors. Our aim was to determine whether such alterations had a prognostic value in Ewing's family tumor (EFT) patients. The *KIF14* and *MDM4* / ***PI3KC*** 2β and the centromeric satellite sequence of chromosomes 8 and 12 were used for replication timing assessments. Aneuploidy was assessed by enumerating the copy numbers of chromosomes 8 and 12. Replication timing and aneuploidy were detected cytogenetically using multicolors fluorescence in situ hybridization assay applied in 135 EFT. Patients with trisomy 8 presented an association with an asynchronous replication pattern (SD) of *MDM4* / ***PI3KC*** 2β genes ( *p* = 0.013). Trisomy 12 was associated with a synchronous pattern (DD) of *KIF14* probe signals ( *p* = 0.04). The DD synchronous replication pattern of *KIF14* showed a correlation with age ( *p* < 0.0001), and the SS synchronous replication pattern of the same locus showed a correlation with lung metastatic ( *p* = 0.012). The subgroup of patients presenting with multiplet signals of *MDM4* / ***PI3KC*** 2β showed an association with treatment response ( *p* = 0.045) and age ( *p* = 0.033). Replication pattern of *KIF14* may, significantly, be associated with chromosomal instability as *MDM4* / ***PI3KC*** 2β may be a considerably new marker of poor treatment response in EFT patients.

## Introduction

The temporal order of allelic replication, whereby both alleles of a gene expressed in a biallelic mode replicate synchronously or where the alleles of a gene undergoing monoallelic expression replicate asynchronously (allele-specific replication), has the expressed allele replicating earlier than the silenced allele. 1 Imprinting, X-inactivation, and allelic exclusion may be the cause of the silencing of a single allele 2 where an unscheduled alteration in replication timing may delay the process of chromosome condensation, increasing genetic instability in several tumor cell lines and primary tumor samples. While the timing of replication has been investigating as an epigenetic marker demonstrating whether a particular gene is programmed to express biallelically or monoallelically, 3 4 others have established the asynchronous allelic replication of various human monoallelically expressed genes, such as those subjected to X inactivation, as well as numerous imprinted genes. 5 Previous published results in the peripheral blood lymphocytes of patients with hematological malignancies or solid tumors—such as renal cell carcinoma or prostate cancer and breast cancer 6 genes that replicate synchronously including tumor suppressor genes *TP53* and *RB1* and oncogenes *C-MYC* and *HER2* lose their synchronous mode and replicate asynchronously.

It is not yet completely understood how this sequential progression of replication in S-phase is regulated in human cells. Disruption of this process can be altered in cells with aneuploidy or a genetic predisposition to cancer 2 7 identifying as a possible marker for genetic instability related to cancer. 8 9 In this way, the allelic synchronization correlated with a higher frequency of aneuploidy is frequently lost in cells derived from patients with various cancers, such as leukemia, 10 in which aberrant chromosomes are known to bypass cell-cycle checkpoints and escape apoptosis 7

Several studies have consistently shown trisomy of chromosomes 8 and 12 as the most common genetic abnormality in EFT patients. 11 12 13 14 15 16 Gains in chromosome number have been associated with more advanced disease in several solid tumors and are thought to reflect the loss of balanced control of mitotic disjunction. 17 Chromosomal aberrations acquired in addition to the *EWS* gene rearrangements have been postulated to serve as markers for more refractory disease. The 1q32.1 region investigated here encompasses two potential oncogenes, *KIF14* and *MDM4/* ***PI3KC*** 2β. KIF14 is an essential protein for cytokinesis and chromosome segregation, 18 and its overexpression is already reported to be associated with poor prognosis in lung cancer 19 , while increased MDM4 levels and the resulting inactivation of p53 contribute to the development of breast cancers. 20

The interphase fluorescence in situ hybridization (iFISH) assay has been used to visualize specific genomic DNA sequences in interphase nuclei, making it possible to accurately examine the replication timing pattern of various loci within a single cell. 2 21 22 23 To the best of our knowledge, none has related the replication pattern of genes expressed in formalin-fixed paraffin-embedded (FFPE) tissue to clinical-pathological parameters of cancer patients so far.

In the current study, we estimated the replication patterns of *KIF14* and *MDM4/* ***PI3KC*** 2β homologous loci in EFT patients and associated them with known significant aberrations such as polysomy 8 and 12 to estimate the influence of these specific aberrations on gene replication. We further examined the relationship between the clinical-pathological features of EFT patients and the behavior of the *KIF14* and *MDM4/* ***PI3KC*** 2β loci.

## Materials and Methods

### Patients and Tumors Specimens

We retrospectively analyzed a total of 135 paraffin-embedded patient tumors retrieved from the pathological files between 1980 and 2008 at the Pathology Department of A.C. Camargo Hospital of Sao Paulo, Brazil. The diagnosis was given by the histopathology review, immunohistochemical staining for CD99 and FLI1 antibodies, and by iFISH for *EWSR1/FLI1* fusion presence or not. In this series, confirmation of 22q12 rearrangements was present in 95.5% of the cases. Thus, the tissue microarray (TMA) slide included 135 cases in duplicate, of which 118 cases were primary tumors, while 17 cases were recurrent tumors. Retrospective clinical data gleaned from the corresponding patients' records included demographic characteristics, surgical resection data, and clinical outcome. Criteria for inclusion were initial treatment of the primary tumor in Hospital A.C. Camargo; clinical data available and FFPE tissue with material sufficient and with quality for the study; and at least 3 years of follow-up (except patients who died of the disease before 3 years). We excluded patients with a second synchronous tumor. Patients' treatment included multiagent chemotherapy, local control measures with radiotherapy and surgery, or a combination of these as appropriate for the period of the study. The most utilized drugs were ifosfamide, cyclofosfamide, adriamicine, etoposide, vincristine, and actinomicine. Radiotherapy was indicated for unresectable tumors, resectable, or partially resectable tumors in patients with metastatic disease and as palliative treatment. 24 Of 135 cases, 104 patients were treated with preoperative treatment and 29 patients were untreated; therapy status was unknown for two patients. The patient's clinical-pathological characteristics are depicted in Table 1 . This study has been reviewed and approved by the Ethical Review Board of A.C. Camargo Hospital.

**Table 1:** Clinical-pathological characteristics of the study patients

Variables	Categories	Patients *N* (%)
Gender	Male	82 (60.7)
	Female	53 (39.3)
Age (0.25–52 y)	S15	74 (54.8)
	>15	61 (45.2)
Primary tumor	Osseous	121 (89.6)
	Extraosseous	14 (10.4)
	Inferior extremity	61 (45.2)
Primary tumor site	Pelvis	31 (23)
	Superior extremity	28 (20.7)
	Thorax	14 (10.4)
	Others	1 (0.7)
Status at diagnosis	Localized	97 (71.8)
	Metastatic	38 (28.2)
Onset of symptoms (0.17–60 mo)	Pain	30 (22.2)
	Swelling	12 (8.9)
	Pain + swelling	61 (45.2)
	Others	24 (17 0.8)
	Ignored	8 (5.9)
Treatment	Treated	104 (77)
	Untreated	29 (21.5)
	Ignored	2(1.5)
Relapse	Yes	69 (51.1)
	No	65 (48.1)
Outcome	Alive without disease	34 (25.2)
	Alive with disease	17 (12.6)
	Death due to disease	83 (61.5)
	unknown	1 (0.7)

#### Probe Selection

**Fig. 1:**
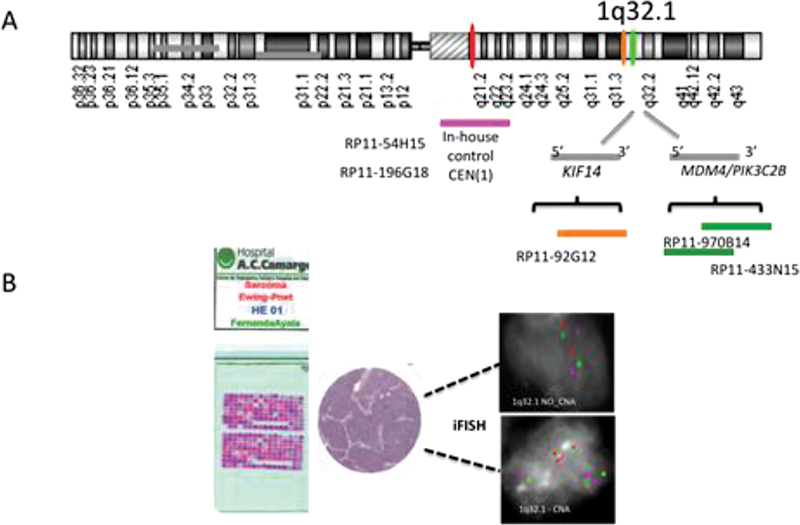
FISH strategy analysis of *KIF14* or *MDM4/ P/3KC28* genes replicating pattern in EFT cells at interphase. ( **A** ) The schematic chromosome 1illustrates the genomic localization and names of the BAC probes encompassing the pericentromeric region and the target genes at 1q32.1. ( **B** ) Representative FISH scheme are shown for EFT TMA applying the three-color FISH for the replication pattern analysis.

The University of California Santa Cruz Human Genome Browser Database, March 2006 build (Greulich-Bode et al, 2008), was used to select the bacterial artificial chromosome (BAC) genomic name clones and their linear order ( www.genome.ucsc.edu ). The strategy for the assessment of copy number alterations (CNAs) in EFT by iFISH was designed using BAC clones (RP11-192G12, RP11-97B14/RP11-433N15) covering approximately 4 Mb of the 1q32.1 cytoband ( *KIF14, MDM4/* ***PI3KC*** 2β) and BAC clones (RP11-54H15/RP11-196G18) for the 1q21 cytoband (pericentromeric region; Fig. 1 ). Subsequently, the BAC clones were obtained from the Centre for Applied Genomics, Sick Kids Hospital (Toronto, Ontario, Canada). Each test probe was paired with a reference probe in either the same chromosome arm or the chromosome centromere to control for numerical changes. Commercial probes (SpAqua -D8Z2 and SpOrange- D12Z3 - Abbott Molecular, IL, United States) were used for the interphase FISH enumeration of the whole chromosomes 8 and 12.

#### Probes Preparation and Fluorescent in situ Hybridization

BAC DNA (1 μg) was extracted by modification of the Qiagen mini-Kit protocol (Qiagen, Valencia, CA, United States) and labeled with SpectrumGreen-dUTP, SpectrumOrange-dUTP (Abbott Molecular), or SpectrumRed-dUTP (PerkinElmer Life and Analytical Sciences, Boston, MA, United States) using the Vysis Nick Translation Kit (Abbott Molecular). Labeling of probes was done as described previously. 25 26 The locus specificity and the correct chromosome location of all BAC clones were confirmed by polymerase chain reaction and metaphase FISH of normal peripheral lymphocytes, respectively.

FISH assays were performed using mixtures of the labeled BAC probes, according to previously published procedures, 27 with slight modifications, the incorporation of the microwave oven for pretreatment with citrate buffer and for crossed-link protein breakage to enhance the hybridization efficiency as described elsewhere. 28 29 Briefly, TMA slides were deparaffinized, dehydrated, and air-dried. Slides were pretreated in sodium citrate (10 mM) using the microwave in three intervals of 5 minutes. Later, the slides were digested with 750 U/mL pepsin (Sigma-Aldrich) at 37 **°** C for 8 minutes. The slides were then washed in 2× saline-sodium citrate (SSC) and dehydrated in a graded ethanol series. Air-dried slides were warmed to 37 **°** C inside the ThermoHybrite (Vysis—Abbott Laboratories), and then the probe mixture was applied to the target DNA area and a cover slip was sealed in place with rubber cement. Slides were placed in the microwave and heated at high power (920 W) for 70 seconds. Slides were codenatured at 80 **°** C for 10 minutes and hybridized in a humidified chamber at 37 **°** C for 16 to 20 hours (ThermoHybrite - Vysis—Abbott Laboratories). Following hybridization the slides were washed at 75 **°** C in 2× SCC/0.3% NP40 for 2 minutes, followed by 5 minutes in 2× SCC at room temperature. Slides were mounted with 4′,6-diamidino-2-phenylindole, dihydrochloride—Vectashield (Vector Laboratories, Inc. Burlingame, CA, United States) and, after 24 hours at −20 **°** C they were examined blindly using an epifluorescence Zeiss Imager. M1 microscope was equipped with a digital camera AxioCam MRm and AxioVision 4.3 capturing software (Carl Zeiss Canada Ltd.).

### Cytogenetic Analysis

#### *KIF14* and *MDM4/PI3KC2β* Locus (1q32.1)

The replication patterns of genes in the 1q32.1 locus were assessed by the configuration of hybridization signals following three-color iFISH with a control probe for the pericentromeric region. 30 We analyzed the relative proportions of the replication pattern of cells in three groups: (1) all populations, (2) those with CNAs, and (3) those without CNAs. For each sample, at least 100 nonoverlapping, intact nuclei exhibiting distinct well-defined fluorescence hybridization signals were scored.

**Fig. 2:**
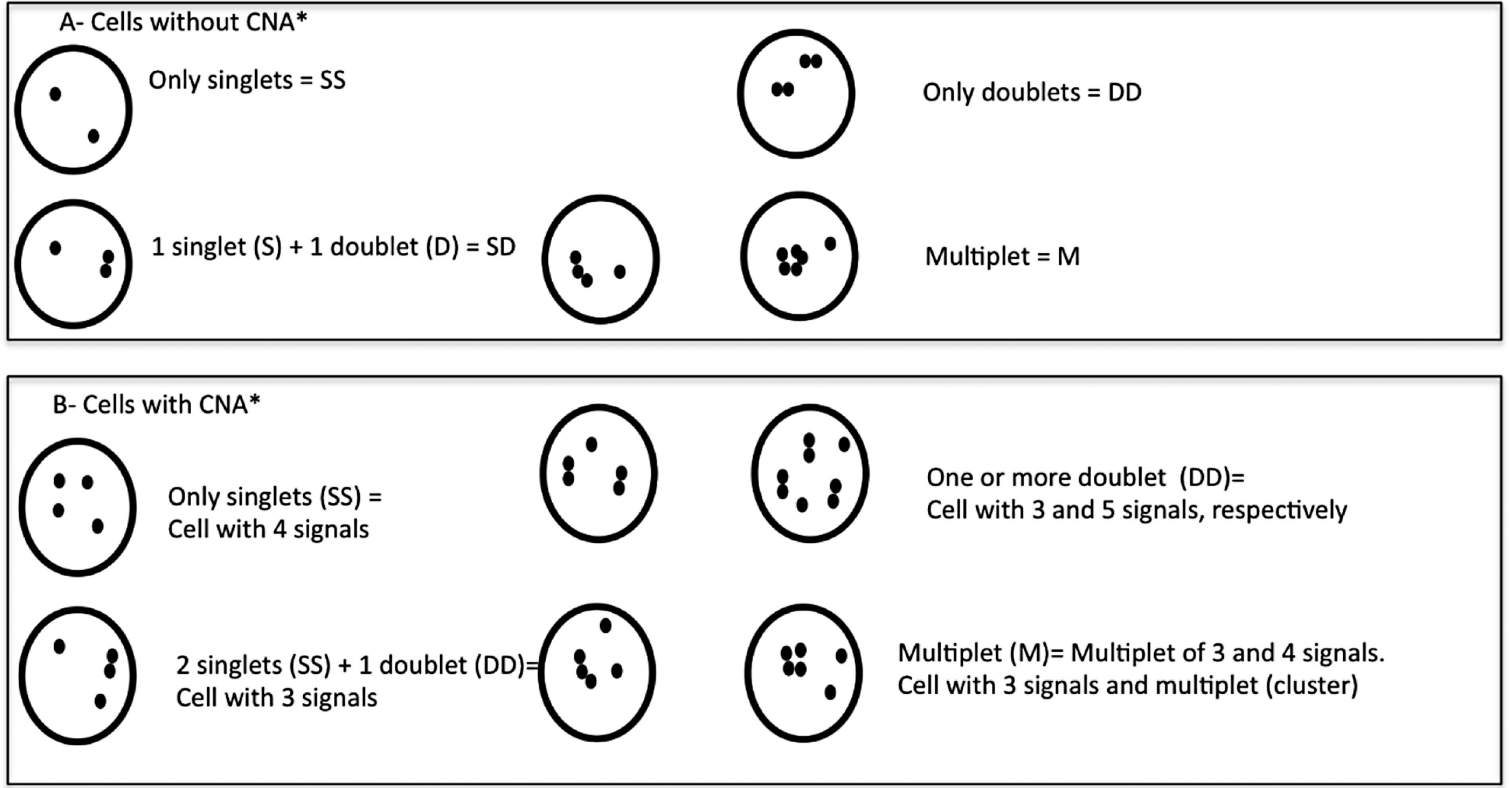
Schematic representation of timing replicating pattern of the *KIF14* or *MDM4/ Pl3KC2B* hybridization signals at interphase by FISH. ( **A** ) Configuration of signals in normal (no CNA) cells showing different replication patterns. ( **B** ) Guide for signal enumeration when the genes dots show up as doublets or multiplet. Note that in cells that showed gain of the genes, only the SS, DD, and M patterns were counted. Abbreviation: CNA*: copy number alteration.

The examined cells were classified into four categories: (1) SS, for cells with two or more similarly shaped signals, or singlets, per locus; (2) DD, for cells with one or more doublets per locus; (3) SD, for cells with one singlet and one doublet signal per locus; and (4) multiplets, for cells with three or more signals spaced less than one signal diameter apart ( Fig. 2 ). We recorded the frequency of SS, DD, SD, and multiplets in each cell population. Thus, based on the rate of the four replication configurations, it was possible to differentiate between three distinct replication patterns of homologous loci as previously described in Selig et al, (1992): (1) a synchronous replication pattern as revealed by two or more singlets (SS) or unreplicated loci in G1; (2) a synchronous replication pattern where cells have two or more doublets (DD) or replicated loci in S or G2; and (3) an asynchronous pattern with a relatively long time interval between early- and late-replicating loci, as indicated by two different signal configurations describing two nonsuccessive replication stages (SD), representing the S-phase where only one allele has been replicated. In cells that showed extragene copies (i.e., CNA), the scoring was done by counting the number of singlet (SS), doublet (DD), and multiplet (M) signals, probably representing an average of several different replication times within a limited range of S phase. 21 It has been demonstrated elsewhere that genes which replicate early in the cell cycle will show a high percentage of doublets, while for late-replicating genes, most nuclei will have singlet hybridization signals. 21 Therefore, it was assumed that the presence of a larger number of doublets represented an earlier time of replication. 7 21

### Detection of Aneusomy

A dual-color iFISH assay was performed here to estimate the rate of aneusomy within cell populations. A conservative analysis of copy number enumeration was measured by counting the copies of chromosomes 8 and 12 based on the cut-off of 10% being level for the assay (mean ± 2 seconds in tumor diploid controls). Hence, we had two groups of genetic parameters – one normal (no CNA) and the other with CNA. For each cell, we recorded the frequencies of cells with one, two, and three or more signals.

### Statistical Analysis

The iFISH data for the gene replication patterns of the 1q32.1 locus were classified as continuous variables for all populations of EFT patients. The parameters included in the analysis were onset of symptoms to diagnosis, age (≤15 vs. >15 years), metastatic location at diagnosis (lung vs. nonlung), treatment response (Huvos grade), trisomy 8 and 12, and gains at 1q32.1. The nonparametric Mann–Whitney test was applied for testing differences between study groups for quantitative clinical parameters. The Spearman correlation was applied for testing the correlation between the different clinical parameters in the study group. A *p* -value of less than 0.05 was considered to indicate statistical significance. All tests were two sided. Statistical analysis was performed using Statistical Packages of Social Sciences (SPSS for Macintosh, Version 16.0, Inc, Chicago, IL, United States).

## Results

Multicolor iFISH was applied to EFT TMA comprising 135 patients by scoring for 100 cells in each tumor core. We examined whether there was a relationship between the replication behavior of genes and clinical-pathological characteristics as described below.

**Fig. 3:**
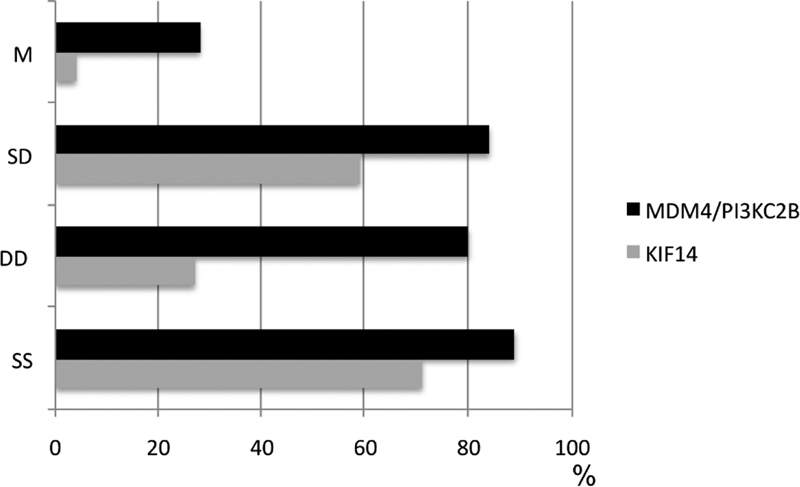
Frequency of cells showing replication timing (SS, DD, SD, M) of *KIF14* and *MDM4/ P/3KC2B* alleles in the total EFT patients.

**Fig. 4:**
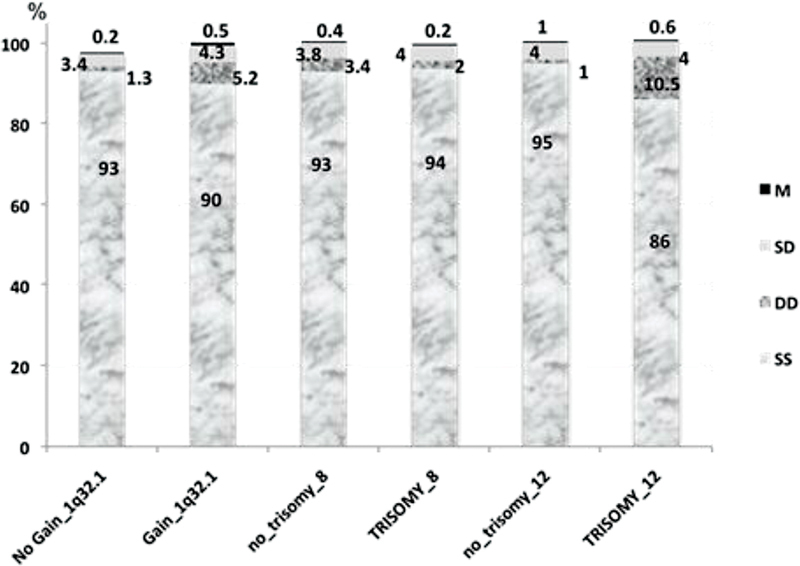
Distribution of cells showing replication timing (SS, DD, SD, M) of KIF14 alleles in EFT patients. The signals were evaluated in cells of EFT patients with CNA and with no CNA for chromosome gains at 1q32.1 and aneusomy of chromosomes 8 and 12.

**Fig. 5:**
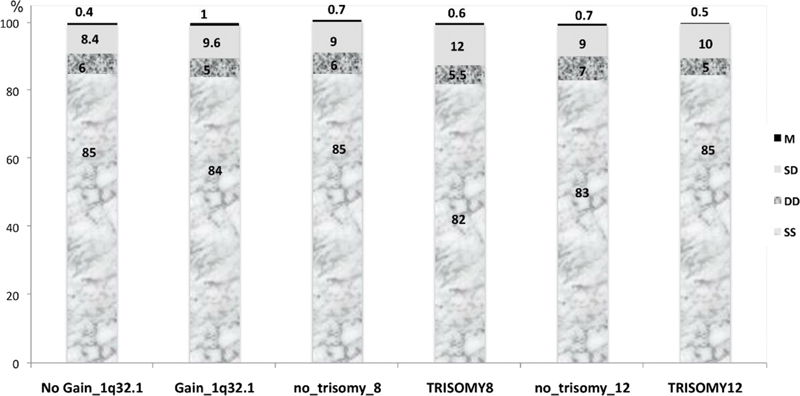
Distribution of cells showing replication timing (SS, DD, SD, M) of *MDM4/P/3KC2B* alleles in EFT patients. The signals were evaluated in cells of EFT patients with CNA and with no CNA for chromosome gains at 1q32.1 and aneusomy of chromosomes 8 and 12.

Different replication timing patterns were evaluated in 90/135 cases for *MDM4/* ***PI3KC*** 2β and in 71/135 cases for *KIF14* in the total population. The proportion of cells with asynchronous (SD and M) replication and synchronous replication (SS and DD) patterns of *KIF14* alleles and of *MDM4/* ***PI3KC*** 2β alleles in the study groups are shown in Figs. 3 , 4 , and 5 . The most frequent replication pattern seen in all populations was the asynchronous stage (SD) either for *KIF14* or for *MDM4/* ***PI3KC*** 2β, varying between 1.4 to 23.2% and 1.1 to 13.6%, respectively. For the group that showed gain at the 1q32.1 locus, there was an increase of SD, DD, and M patterns for *KIF14* alleles, whereas for *MDM4/* ***PI3KC*** 2β signals, the SD and M replication patterns were increased when compared with the group that did not showed gain at that region. The higher frequency of the asynchronous pattern for *MDM4/* ***PI3KC*** 2β indicates that, in EFT cells, its two loci differ largely in their replication timing. Additionally, in the group with trisomy 8, there was a slightly higher rate of the SS synchronous pattern for the *KIF14* and *MDM4/* ***PI3KC*** 2β alleles than in the group with trisomy 8. Furthermore, in the group with trisomy 12, there was a higher rate of DD synchronous pattern for the *KIF14* alleles, whereas for the *MDM4/* ***PI3KC*** 2β alleles, there was a slight increase of the SD pattern compared with the group without trisomy 12. Also, there were more cells with single dots in the studied groups, but without statistical significance. The difference in the proportion of cells with SD pattern in the groups studied (1q32.1, trisomy 8 and 12) did not show a statistically significant correlation for both probes analyzed. Interestingly, there was a significant negative correlation between SS and DD, SD, and M patterns for both hybridization signals for *KIF14* and *MDM4/* ***PI3KC*** 2β ( *p* < 0.05) in the total population.

### Statistical Analysis of Replication Pattern and Clinical-Pathological Features

The group of patients with trisomy chromosome 8 (27/124) presented a statistically significant association with cells that showed an asynchronous (SD) replication pattern of *MDM4/* ***PI3KC*** 2β genes ( *p* = 0.013). Also, the subset of patients with trisomy chromosome 12 (18/124) showed a statistically significant association with a synchronous (DD) pattern of *KIF14* probe signals ( *p* = 0.04) in all populations.

The DD synchronous replication pattern of *KIF14* showed a correlation with age ( *p* < 0.0001), whereas the SS synchronous replication pattern of the same locus showed a correlation with the metastatic site (lung vs. nonlung; *p* = 0.012). Our results also showed a negative correlation between the DD synchronous replication of *KIF14* and the onset of symptoms ( *r* = −0.260, *p* = 0.03). Additionally, the subgroup of patients presenting with the multiplets replication pattern of *MDM4/* ***PI3KC*** 2β genes showed an association with clinical response ( *p* = 0.045) in all populations and in the group with gain at 1q32.1 (15/128). Thus, patients who had poor treatment response (Huvos grade I, II 31 ;) showed a lower mean (0.29 vs. 1.0) of multiplet signals per cell. This multiplet replication pattern of *MDM4/* ***PI3KC*** 2β genes showed an association with age ( *p* = 0.033) in all populations.

## Discussion

Ewing family tumors (EFT) are highly aggressive cancers for which drug resistance and relapse remain a constant clinical dilemma. Also, the cellular mechanism that underlies the difference between nonresponders and those who respond to treatment is still unclear. Here, we observed that the polysomy of chromosomes 8 and 12 and the clinical-pathological characteristics of EFT patients are related to the replication behavior of alleles of *KIF14* and *MDM4/* ***PI3KC*** 2β loci.

**Fig. 6:**
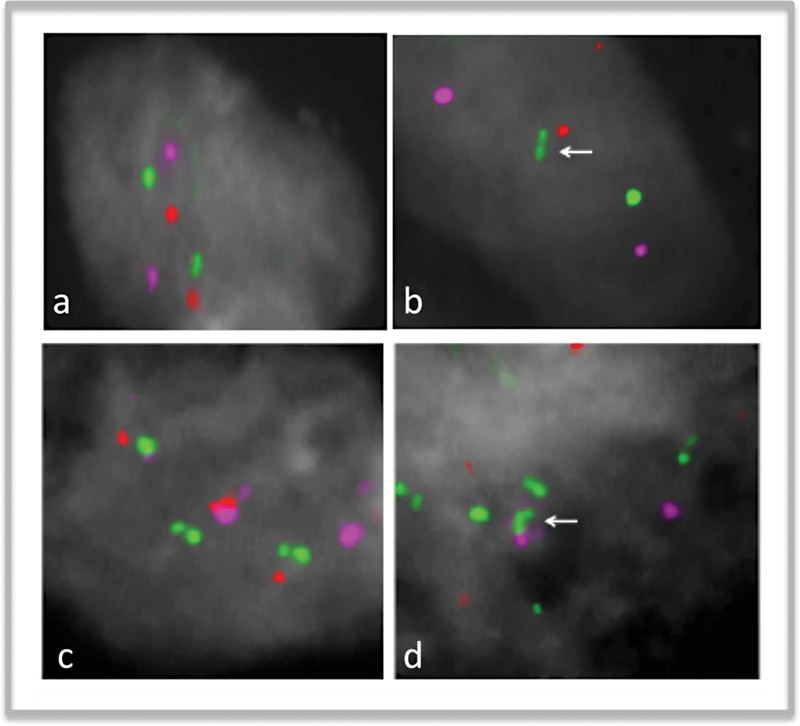
Representation of FISH images of 1q32.1 region showing replication pattern in EFT TMA cells at interphase applying the three-color iFISH assay. *MDM4/P/3KC2B* signals hybridization are seen in green. *KIF14* signals hybridization are seen in orange and the control in *pink.* ( **A** ) cell with singlets (SS cells) in which neither allele has replicated; Synchronous pattern ( **B** ) cell with one singlet and one doublet (SD cells), which are Sphase cells in which one allele has replicated while its partner has not ( *arrow* ), ( **C** ) cell with doublets (DD cells) in which alleles have replicated; and ( **D** ) cell with multiplets pattern of signals (arrow).

The iFISH replication assay used here has already been proved sufficiently reliable for comparing the replication status of one gene homolog relative to its counterpart in the same cell 21 22 32 33 ( Fig. 6 ).

We reported in the current study that the most frequent replication pattern seen in all cell populations was the asynchronous stage (SD), suggesting a loss of control of replication timing in EFT cells. Moreover, the two pairs of allelic *KIF14* and *MDM4/* ***PI3KC*** 2β loci replicated highly asynchronously in the group that showed gain than in the group with the absence of aberrations at 1q32.1. This higher frequency hemizygous replication pattern is probably a consequence of the genes' rearrangement in these EFT cells. Similar to, the asynchronous replication pattern of the *c-MYC* gene seen in lymphoma cells, in which the rearranged 5′ end replicates later than the unaltered copy (Hayday et al, 1984; Calza et al, 1984). However, the allele-specific epigenetic alteration leading to functional hemizygosity of several genes within a single cell may also create a situation where a dominant oncogene is expressed in cells in which it is normally not expressed (Crossen, 1997), unlike the mechanism of LOH, which causes silencing of tumor suppressor genes. Histone acetylation and deacetylation states related to DNA methylation are also altered during gene regulation to affect transactivation of genes (Coolen et al., 2010). Yet, these genes may be located in the transition points between early- and late-replicating domains where some fork barriers and repetitive elements are hot spots for DNA breaks and chromosome rearrangements 34 35 36 and, consequently, may be more prone to changes in gene sequence and in replication timing.

In the group that showed gain at 1q32.1, we noted that both *KIF14* alleles had a rate of SS patterns lowered with a corresponding increase in the rate of SD, DD, and M patterns, meaning that at least one allele is early replicating compared with its normal replication schedule in some cells. For *MDM4/* ***PI3KC*** 2β hybridization signals, we found a prevalence of the synchronous SS replication in the group without CNA but an increase in the SD and multiplet pattern with gain at 1q32.1 ( Fig. 2 ), implying a loss of replication control in the cell cycle, which may lead to an overexpression of *MDM4/* ***PI3KC*** 2β in these cells. We demonstrated the existence of a high variability of cell-cycle phase among different EFT patient groups with CNA or without CNA. On the contrary, when the replication timing is highly synchronous (SS and DD), it has been indicated that a largely diploid chromosomal constitution is present (Selig et al., 1992).

Moreover, the polysomy of chromosome 8 showed significant statistical association with the SD replication pattern of *MDM4/* ***PI3KC*** 2β, while the polysomy of chromosome 12 was statistically associated with the DD replication pattern of *KIF14* for all populations. This may indicate a concentration-dependent requirement for KIF14 protein in chromosome disjunction, and failure of this association could contribute to cancer progression. Accordingly, other groups' data show that alteration of the replication pattern of chromosome regions may be one of the manifestations of genetic instability associated with increased risk for transformation. 7 37 We also observed a higher rate of asynchrony (SD) of the *MDM4/* ***PI3KC*** 2β locus in the trisomy 8 and 12 group compared with EFT patients without such aberrations, a finding which could reflect a correlation between the chromosomal aberration and gene replication leading to loss of cell-cycle control, as happen in chronic lymphocytic leukemia patients who presented with trisomy 12. 7

It is clear that the EFT patients' cells show a high variability of replication pattern in normal (no CNA) and aberrant cells (with CNA) during the cell cycle, and the pattern that we detected in tumors with gain at 1q32.1 represents an average estimate among the cells. Furthermore, our results indicated that the relationship between replication pattern and clinical-pathological characteristics could be transmitted in the behavior of the genes' loci (1q32.1). Regarding our statistical results, there was a correlation between the synchronous (SS) *KIF14* replication pattern and lung metastasis and age. Evidence has indicated that tissue-specific genes replicate early in the cell cycle and late in tissues in which they are silent. Although previous study indicates *KIF14* as a molecular signature of poor prognosis in lung carcinoma, in our results linking the gene to lung metastasis, we demonstrate a late replication pattern, suggesting this gene locus must have undergone a switch in replication timing in an expressing cell type. 36 38 Besides, based on *in silico* analysis, 39 the entire *KIF14* gene was covered by a copy number variation region. In this manner, late replication due to inherent difficulties of replicating DNA could be a result of an accumulation of ssDNA that is more susceptible to damage and lost of control replication. 35 Additionally, in our findings, the early *KIF14* allelic expression correlates with patients younger than 15 years of age. These younger patients had more DD replication pattern of *KIF14* (mean 1.8 vs. 0.38) than patients older than 15. The DD synchronous pattern could be an indication of premutated genes. 40 Hence, we infer that in younger patients premutations may be an early event in EFT tumorigenesis, as they may possess a higher actively replication mode needed for the endogenous events and cell-cycle progression and more aggressive disease phenotype. Moreover, the negative correlation of time between onset of symptoms to diagnosis and synchronous replication pattern (DD) of the *KIF14* locus suggests that patients who tolerate the symptoms longer have a lower rate of the DD pattern and potentially lower expression of *KIF14,* which is present during cell cycle. Noteworthy to say that no correlation was found between the treated or untreated group and the replication pattern of genes targeted at 1q32.1 locus. This is in line with a recent study that shows an abnormal pattern of replication (an epigenetic aberration) in lymphocytes with hematological disease at diagnosis is the same as that observed after chemotherapy but absent following allogeneic stem-cell transplantation. However, alteration in aneuploidy (a genetic aberration) that accompanies hematological malignancies, in contrast to epigenetic aberration, does not disappear following transplantation. 6 41 Conversely, within the treated group, we found different replication patterns between the treatment responders and nonresponders with the *MDM4/* ***PI3KC*** 2β multiplet replication pattern (an asynchronous pattern). Patients who had poor treatment response showed lower frequency of the multiplet replication pattern of *MDM4/* ***PI3KC*** 2β genes. In non-Hodgkin lymphoma, asynchronous allelic replication is indicative of higher risk of relapse 7 and allelic-replication mode is a more accurate novel blood marker than the PSA antigen level in peripheral blood lymphocytes to differentiate between prostate cancer and benign prostate hyperplasia 2 . These findings led to the prospect of the *MDM4/* ***PI3KC*** 2β multiplet replication pattern as a new genotoxic marker for different EFT nonresponder genotypes with a transcriptional and cell-cycle deregulation.

Following this line of thought, there is a notion that the association between the *MDM4/* ***PI3KC*** 2β multiplet replication pattern and clinical response may be related to chemotherapy agents that can induce hypomethylation of DNA, which is the mechanism whereby genotoxic agents affect timing gene replication (Haaf, 1995) not discarding the possibility of being a reversible event after a stem cell transplantation, as described. 41 In this respect, it is possible to build a model that is consistent with our data—cytotoxic agents may bind to transcription factor DNA-binding domains, which attach to specific sequences of DNA adjacent to the genes that they regulate and leading to cell survival. Thus, this complex will control the aberrant replication timing and transcription for the transference of genetic information from DNA to mRNA. This is in line with the large amount of data showing correlation between allelic-replication timing and gene-expression profiles. 1 23 33 36 42 We suppose that in EFT patients, therapy resistance and eventual relapse can be triggered by aberrant regulation of the *MDM4/* ***PI3KC*** 2β transcripts leading to escape from apoptosis, and therefore, tumor growth. Also, patients with worse treatment response have less multiplet hybridization signals 42 ; however, they may have a more intricately disorientated molecular complex during the cell-cycle and DNA damage response. They may not only have deregulated activity of the DNA and RNA polymerases, but also may have more efficient DNA repair and cell-cycle checkpoint pathways leading to a deregulation of the DNA repair mechanism. Consequently, EFT tumors may survive due to generation of heterogeneous clonal cell populations.

Taken together, we have seen here a modified order of allelic replication in EFT patients at different genetic and clinical-pathological EFT groups, from which we suggest that the average of multiplet replication pattern of *MDM4/* ***PI3KC*** 2β may be an indicator of poor clinical response in EFT and the replication pattern of *KIF14* may contribute to chromosomal instability in EFT patients. We feel that an cell survival could result from underlying epigenetic process in combination with genetic mechanisms that could arise from structural effects of drug/radiation therapy on DNA, deregulating replication, and transcription. Additionally, patients that respond poorly to treatment might have more efficient DNA damage repair pathways to escape from apoptosis than the responsive patients. Naturally, more experiments will be needed to confirm the intriguing correlations that we found between replication pattern and clinical-pathological features to extend the consistency of these data in EFT patients.
